# A novel mitochondrial-related gene signature for assessing the tumor immune microenvironment and predicting prognosis in human glioma

**DOI:** 10.1007/s12672-026-05494-z

**Published:** 2026-06-23

**Authors:** Zakia Harmak, Oumayma Naji, Hamza Baddi, Bouchra Ghazi, Abdallah Badou

**Affiliations:** 1 Immuno-Genetics and Human Pathology Laboratory, Faculty of Medicine and Pharmacy, University Hassan II (UH2C), Casablanca, Morocco; 2 Immunopathology-Immunotherapy-Immunomonitoring Laboratory, Faculty of Medicine, Mohammed IV University of Sciences and Health (UM6SS), Casablanca, Morocco; 3 City of Innovation and Technology Transfer, University Hassan II (UH2C), Casablanca, Morocco

**Keywords:** Mitochondrial-related gene, Gene signature, Tumor immune microenvironment, Glioma

## Abstract

**Background:**

Gliomas represent the most prevalent and aggressive primary brain tumors in adults. Accumulating data suggest a strong connection between mitochondrial dysfunction and cancer development. However, signature markers for assessing mitochondrial genetic risk in human glioma remain limited. This study aims to identify mitochondrial genes associated with glioma patient prognosis, develop a strong mitochondrial-related gene signature (MRGS), and analyze the tumor immune microenvironment (TIME) related to this signature. The expression profiles and prognostic value of MRGS were analyzed using R language, GraphPad Prism 8 and online databases.

**Results:**

A signature of seven genes including ACSL1, NOX4, ALDH3A2, ALKBH4, FASTK, FDX1, ACADVL was constructed using the TCGA cohort and further validated in an independent CGGA cohort. Our result showed that the mitochondrial signature was significantly associated with high-grade and isocitrate dehydrogenase (IDH) wild-type gliomas, aggressive molecular and histological features, and poor survival. Subgroup analyses demonstrated that the risk signature was significantly associated with overall survival (OS) across WHO tumor grades and IDH mutation status, suggesting added prognostic value beyond established clinical markers. Additionally, the risk signature demonstrated a significant difference in immune cell infiltration between the high-risk (H-R) and low-risk (L-R) groups. Moreover, the risk score was strongly correlated with tumor microenvironment (TME)-related marker and immune checkpoint molecules such as (PD-1, PD-L1, CTLA-4, LAG-3, TIM-3, BTLA, HVEM, NR2F6), suggesting that an immunosuppressive TME could lead to a poor prognosis in H-R groups. Additionally, Multivariable Cox analysis showed that risk score was an independent predictor of poor prognosis in both TCGA (Hazard Ratio (HR) = 2.17, 95% confidence interval (CI): 1.44–3.3, *p* < 0.001) and CGGA (HR = 2.07, 95% CI: 1.49–2.88 *p* < 0.001) datasets. Finally, enrichment analysis highlighted the association of the signature with biological mechanisms involved in tumor progression.

**Conclusion:**

Our findings suggest a novel mitochondrial-related gene signature for the TIME that could be used as a reliable prognostic biomarker for patients with glioma.

**Supplementary Information:**

The online version contains supplementary material available at 10.1007/s12672-026-05494-z.

## Introduction

Gliomas are the most aggressive brain tumors which originate from glial cells of the central nervous system (CNS) and are known for their rapid progression and invasiveness with Glioblastoma (GBM) being the most common and malignant subtype. GBM represents around 57% of all gliomas and 48% of primary fatal brain tumors [1, 2]. The World Health Organization (WHO) classifies glioma into low and high grade glioma and divides it into four subtypes: oligodendroglioma, astrocytoma, glioblastoma, and mixed gliomas [3].

Metabolic reprogramming in the TME and mitochondrial dysfunction in Tumor-Infiltrating Lymphocytes (TILs), have been involved in the impaired immune antitumor activity [4, 5]. On the one hand, mitochondria contribute to the immunosuppressive microenvironment by modulating oxidative phosphorylation and Reactive Oxygen Species (ROS) balance in immune cells, namely effector T cells and macrophages [6]. On the other hand, it has been shown that GBM cells exhibit hyperpolarized mitochondrial membrane potential, allowing cancer cells to escape apoptosis [7]. In addition, the abnormal accumulation of mitochondrial metabolites, such as succinic acid, fumaric acid and 2-hydroxyglutaric acid, and the impairment of the mitochondrial permeability transition function lead to the development of malignant precursors [8–10]. Additionally, it has been shown that mitochondrial dysfunction was found to promote treatment resistance [11].

Considering the relevance of mitochondria in carcinogenesis, numerous Mitochondrial-Related Genes (MTRGs) have been identified as critical driver of cancer development [12]. Such genes impact important mitochondrial activities, including oxidative phosphorylation, apoptosis and metabolic reprogramming, which all promote tumor cell survival and proliferation [13]. Advances in bioinformatics and multi-omics integration have shed new light on cancer progression mechanisms and precision medicine approaches [14, 15]. However, few studies have investigated mitochondrial-related gene signatures in glioma, prompting our study to uncover new prognostic biomarkers.

In this study, we aimed to develop a model based on MTRGs and generate a prognostic risk signature for forecasting the clinical outcome of glioma patients. By using RNA-sequencing data from the Cancer Genome Atlas (TCGA) and Chinese Glioma Genome Atlas (CCGA) databases in combination with bioinformatics and statistical methods, we constructed a seven gene-signature prognostic model using Lasso Regression. The novel mitochondria-related gene model includes ACSL1, NOX4, ALDH3A2, ALKBH4, FASTK, FDX1, ACADVL. We further explored its association with immune cell infiltration, immune checkpoints (ICPs), and clinical outcomes in glioma patients, highlighting the potential interplay between mitochondrial function and tumor immune landscape.

## Materials and methods

### Data origin and gene collection

RNA-sequencing data and corresponding clinical parameters were obtained from the TCGA databases (lgg_tcga_pancancer) and (gbm_tcga_pan_cancer) (https://www.cbioportal.org/) and the CGGA database (https://www.cgga.org.cn/). A total of 674 glioma samples from the TCGA dataset were used as the training cohort to construct the prognostic model, while 325 glioma samples from the CGGA dataset were used as an independent external validation cohort to assess the strength and predictive performance of the MRGS. In the TCGA cohort, low grade glioma (LGG) and GBM samples were first log2-transformed and then quantile-normalized to ensure comparability across samples. Batch effect correction was subsequently performed using the “removeBatchEffect” function from the limma R package (R version 3.6), with batch defined according to dataset origin (LGG vs. GBM). The CGGA dataset was processed using the same normalization strategy to ensure consistency across cohorts. All subsequent analyses, including risk score calculation and model validation, were performed on TCGA and CGGA datasets processed using the same normalization and preprocessing pipeline. Patients were stratified using the median cutoff, a commonly employed approach, with subgroup analyses showing added prognostic value. The panel of 64 MTRGs was selected based on current literature and their functional significance for mitochondrial metabolism and glioma biology, aiming for a focused analysis rather than a broader database such as MitoCarta 3.0.

### Screening of differentially expressed MTRGs

Differentially expressed MTRGs were identified from TCGA and CGGA cohorts, respectively, with false discovery rate (FDR) < 0.05 and logFC > 1. The DGE was analyzed by “limma” package (R v3.6) [16]. Differentially expressed genes (DEGs) were compared between TCGA and CGGA tissue using volcano plots. Overlapping differentially expressed MTRGs between two cohorts were used for subsequent analyses using “venn diagram” package. To exhibit the connection between these genes, a correlogram was drawn using the “corrplot” package [17].

### Development and verification of prediction signature

The Kaplan–Meier method with the log-rank test was used to screen differentially expressed MTRGs associated with the prognosis of glioma patients. Subsequently, LASSO Cox regression analysis was performed using the “glmnet” R package to develop a prognostic signature. A 10-fold cross-validation approach was applied to determine the optimal penalization parameter lambda (λ) based on the minimum cross-validated error, thereby reducing the risk of overfitting. The mitochondrial gene signature was established using genes with non-zero coefficients at the optimal λ value. The risk score for each patient was calculated as the sum of the expression levels of the seven genes weighted by their corresponding coefficients: risk score = Σ (gene expression × coefficient). After constructing the prognostic model in the TCGA training cohort, the same risk score formula was applied to the CGGA cohort for validation. Model performance was assessed using Kaplan–Meier survival analysis, multivariate Cox regression, and time-dependent ROC curves, demonstrating the prognostic value of the MRGS in independent cohorts. To assess potential multicollinearity among variables included in the multivariate Cox regression model, variance inflation factor (VIF) analysis was conducted. VIF values were calculated for all covariates, and a threshold of VIF < 5 was considered indicative of no significant multicollinearity. To evaluate the incremental prognostic value of the risk score, a hierarchical Cox proportional hazards regression analysis was performed. A baseline model including established clinical variables (tumor grade and IDH mutation status) was first constructed, after which the risk score was added to assess its additional contribution. Model improvement was evaluated using the likelihood ratio test by comparing nested models. A significant improvement in model fit following inclusion of the risk score was interpreted as evidence of added prognostic value beyond conventional clinical variables. Calibration curves were generated to assess the agreement between predicted and observed survival probabilities in both the TCGA and CGGA cohorts. In addition, each gene was analyzed individually using Kaplan–Meier survival analysis in GraphPad Prism 8.

### Subgroup analysis of clinical characteristics

To confirm the association between signature and clinical features, a subgroup analysis was performed using TCGA cohort. According to available clinical data, patients were divided into several subgroups, including age (≤ 45 and > 45 groups), gender (male and female groups), tumor grade (Grade II, grade III and IV), histological subtype, IDH status (Wild-type and mutant), and 1p19q codelation (Codel and no codel).

### Immunity analysis

Correlations between stromal and immune cell scores and the risk score were evaluated in glioma patients using gene expression data and the ESTIMATE algorithm. Immune cell infiltration was further assessed using both the CIBERSORT algorithm and the ImmuneCellAI package in R (v3.6). CIBERSORT was applied using the LM22 signature matrix to estimate the relative proportions of 22 immune cell types from bulk RNA-sequencing data, with batch correction enabled (B mode) and 100 permutations performed for statistical significance (1,000 permutations yielded consistent results) [18]. ImmuneCellAI was used to quantify immune cell subsets, particularly T-cell populations [19]. The integration of these complementary methods provided a comprehensive characterization of glioma TIME. Finally, associations between immune cell fractions, immune checkpoint expression, and the risk score were evaluated using Spearman correlation analysis. All analyses were conducted using default or recommended parameter settings for each algorithm to ensure reproducibility and comparability between datasets.

### Gene set enrichment analysis (GSEA)

GSEA analysis was done to identify metabolic pathways and biological processes differentially enriched between the H-R and L-R groups defined by our 7-gene mitochondrial-related signature. The GSEA analysis was conducted using the GSEA software (GSEA 4.33) developed by the Broad Institute. Genes were ranked based on their differential expression between the H-R and L-R groups using the signal-to-noise ratio. Selected gene sets from *Hallmarks*: h.all.v2026.1. Hs.symbols.gmt, *Curated*: c2.all.v2026.1 .Hs.symbols.gmt, *Gene ontology*: c5.all.v2026.1 .Hs.symbols.gmt, *Immunological* : c7.all.v2026.1 .Hs.symbols.gmt were used as reference gene setsAn enrichment was performed with 1,000 permutations to evaluate the statistical significance of each gene set. The normalized enrichment score (NES) was calculated to assess the degree of enrichment, and the FDR was used to correct for multiple hypothesis testing. Gene sets with |NES| > 1, a nominal p-value < 0.05, and an FDR < 0.25 were considered significantly enriched.

### Comparative analysis

To address the comparative performance of our MRGS, we implemented previously published mitochondrial gene signatures in glioma [20] using their original formulas and applied them to the same TCGA and CGGA cohorts. Risk scores were calculated according to the reported coefficients, and their prognostic performance was evaluated using time-dependent ROC curves, concordance index (C-index).

### Statistical analysis

Statistical analyses, graphical representations, and heatmap visualization were performed using GraphPad Prism 8.0.1 and RStudio software (v3.6.3). Relevant R packages and their versions are provided in Supplementary Table.S1 to ensure reproducibility. OS differences between groups were assessed using Kaplan–Meier survival analysis with the log-rank test. Patients were stratified into H-R and L-R groups based on the median risk score derived from the TCGA training cohort, which was consistently applied to the CGGA validation cohort. For group comparisons, the Mann–Whitney test was used for unpaired analyses. Correlation analyses were performed using Spearman’s correlation coefficient. P-values were adjusted for multiple testing using the Benjamini–Hochberg method in high-dimensional analyses, including gene-level and pathway-level comparisons, to control the false discovery rate (FDR). A two-sided p-value < 0.05 was considered statistically significant and statistical thresholds were standardized across all analyses.

## Results

### Identification of differentially expressed MTRGs

**Fig. 1:**
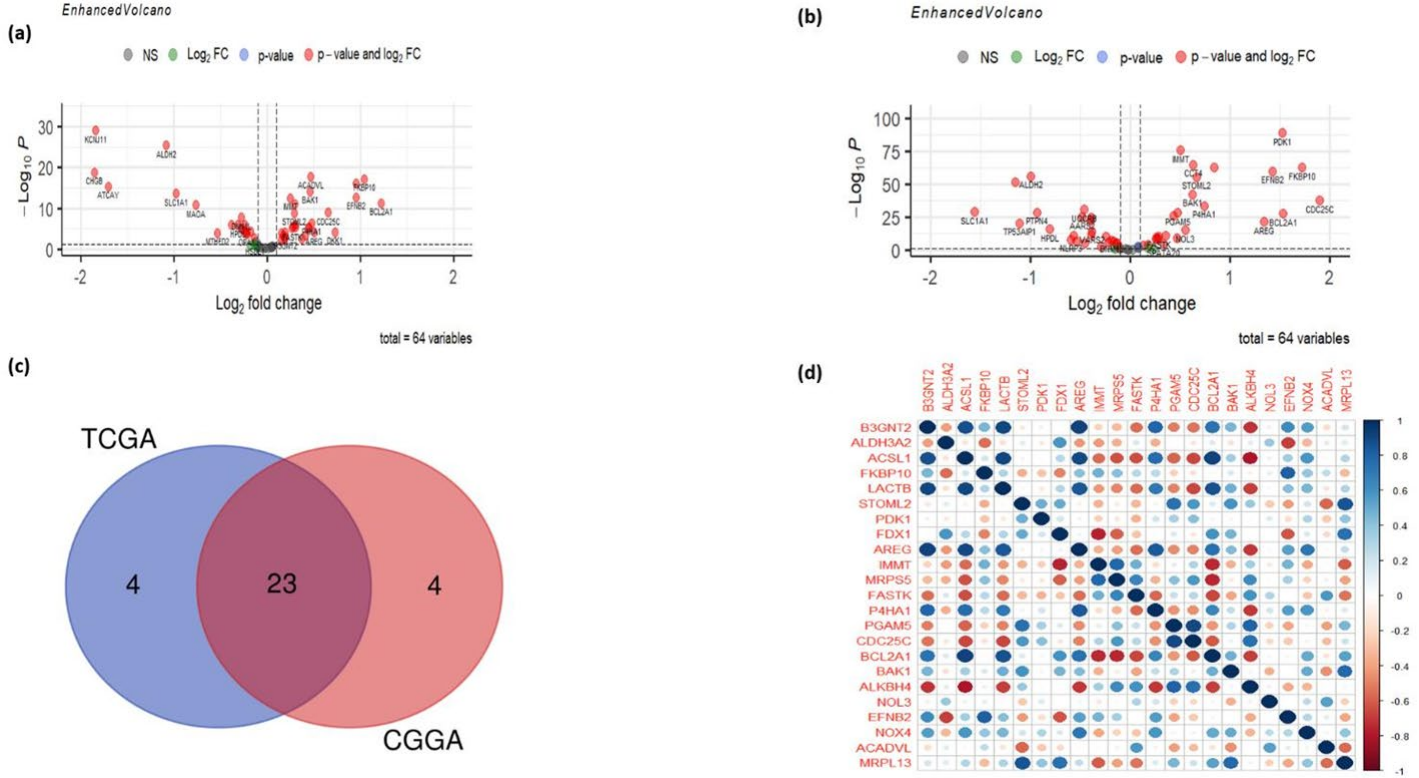
Identification of overlapping differentially expressed MTRGs in TCGA and CGGA cohorts. **a** Volcano plot of differentially expressed MTRGs between low-grade and high-grade glioma in the TCGA cohort. **b** Volcano plot of differentially expressed MTRGs in the CGGA cohort using the same analytical pipeline. **c** Venn diagram illustrating the overlap of differentially expressed MTRGs between TCGA and CGGA, identifying 23 common genes used for subsequent analyses (Supplementary Table S2). **d** Correlation network of the 23 differentially expressed MTRGs based on Spearman correlation analysis. Node size reflects correlation strength and color indicates direction (blue: positive; red: negative)

Among the 64 MTRGs, 27 genes were differentially upregulated in the TCGA cohort (Fig. 1a) and 27 in the CGGA cohort (Fig. 1b). A total of 23 differentially expressed MTRGs were identified as overlapping in GBM across both cohorts (Fig. 1c). Most of these 23 genes were interrelated and their corresponding heatmap is shown in Fig. 1d.

### Prognostic significance of the 23 MTRGs

**Fig. 2:**
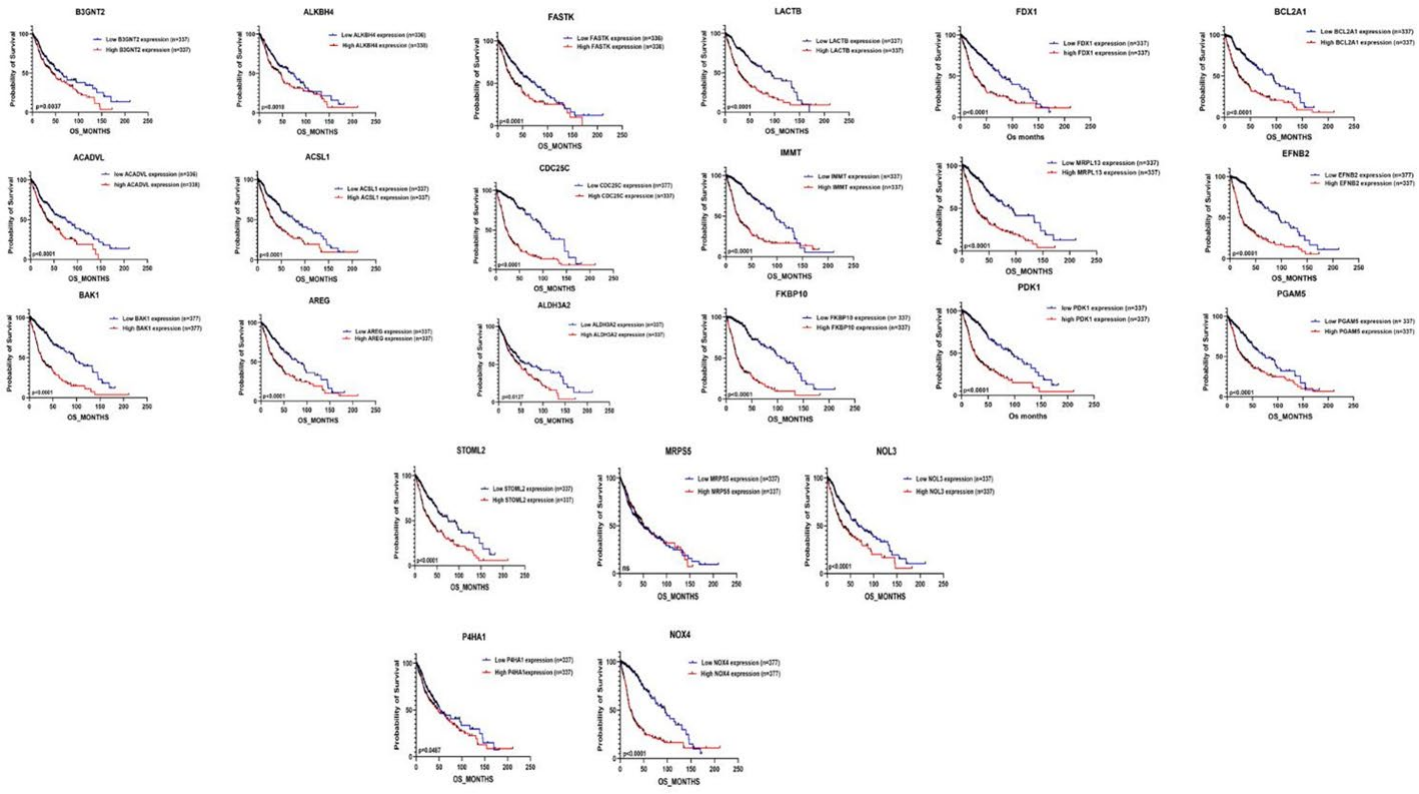
Kaplan–Meier survival analysis of MTRGs in the TCGA cohort. Patients were devided into high- and low-expression groups based on median gene expression. OS differences were assessed using the Kaplan–Meier method and log-rank test. Blue curves represent low-expression groups and red curves represent high-expression groups. “n” indicates the number of patients in each subgroup

**Fig. 3:**
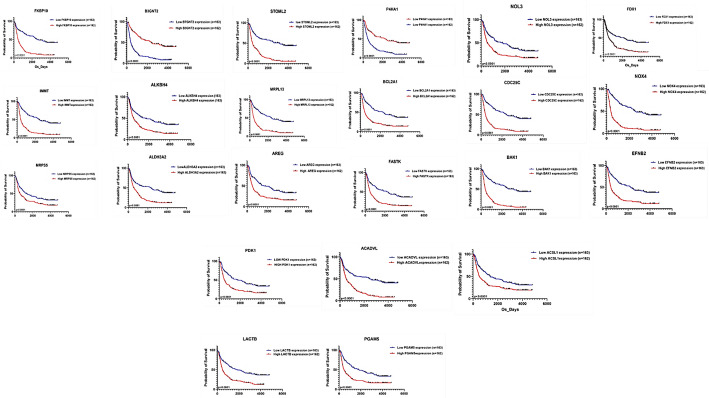
Kaplan–Meier survival analysis of MTRGs in the CGGA cohort. Patients were devided into high- and low-expression groups based on median gene expression. OS differences were assessed using the Kaplan–Meier method and log-rank test. Blue curves represent low-expression groups and red curves represent high-expression groups. “n” indicates the number of patients in each subgroup

Kaplan–Meier survival analysis revealed that 22 genes were associated to poor survival prognosis in the TCGA cohort (*p* < 0.05), including B3GNT2, BCL2A1, ACADVL, ALDH3A2, ACSL1, BAK1, MRPL13, FKBP10, LACTB, ALKBH4, STOML2, PDK1, P4HA1, NOL3, FDX1, AREG, PGAM5, EFNB2, IMMT, CDC25C, NOX4, and FASTK (Fig. 2). In the CGGA cohort, 23 genes were significantly associated to poor survival prognosis (*p* < 0.05) including, B3GNT2, BCL2A1, ACADVL, ALDH3A2, ACSL1, BAK1, MRPL13, FKBP10, LACTB, ALKBH4, STOML2, PDK1, P4HA1, NOL3, FDX1, AREG, PGAM5, EFNB2, IMMT, CDC25C, NOX4, FASTK and MRPS5 (Fig. 3). In fact, the group of patients with high gene expression showed poorer OS than those with low levels. This illustrates the prognostic impact of these genes in predicting unfavorable clinical outcomes and negatively affecting overall glioma survival.

### Development and verification of predictive signature

**Fig. 4:**
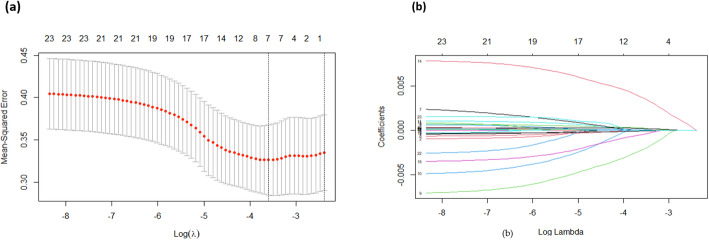
Signature construction. Feature selection was performed using the LASSO regression model with 10-fold cross-validation to identify the most prognostically relevant genes. The optimal tuning parameter (λ) was determined based on the minimum criterion. **a** Coefficient profile plot showing the trajectories of gene coefficients against the log(lambda) sequence. Each curve represents a candidate gene, illustrating how coefficients shrink toward zero as (λ) increases. **b** Cross-validation curve used to determine the optimal lambda value, leading to the selection of 7 genes for construction of the prognostic signature

**Fig. 5:**
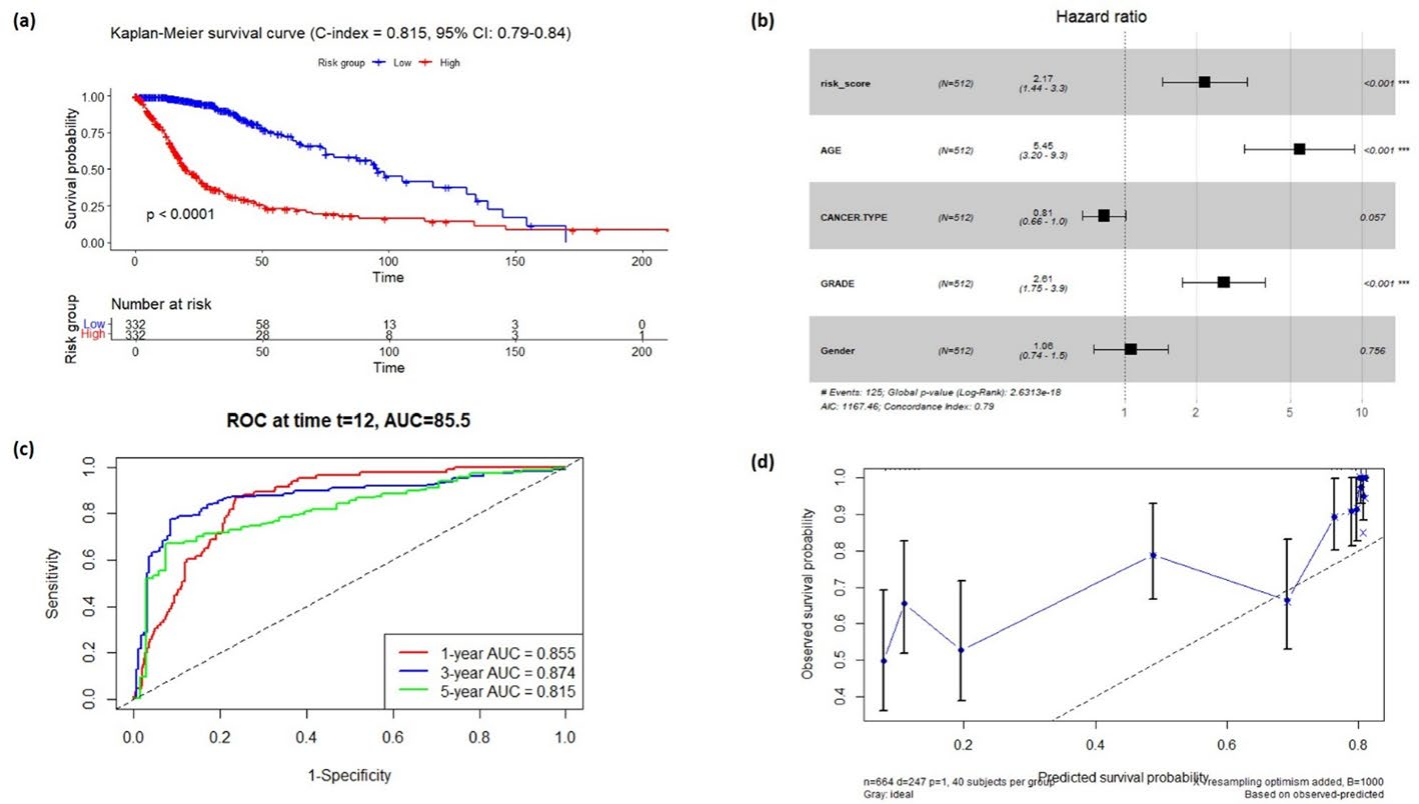
Performance of the prognostic signature in the TCGA cohort. **a** Kaplan–Meier survival curves comparing H-R and L-R groups based on the median cutoff. Blue curve represents the L-R group, and red curve represents the H-R group. “n” indicates the number of patients in each group. **b** Multivariate Cox regression analysis of prognostic factors. The risk score was included as a covariate. A HR greater than 1 indicates that higher risk score is associated with poorer prognosis. (full statistical details in Supplementary Table S3). **c** Time-dependent ROC curves at 1-, 3-, and 5-year for OS. **d** 1-year calibration plot demonstrating agreement between predicted and observed survival probabilities. The dashed line represents perfect calibration, while the blue curve corresponds to the bootstrap-corrected estimates (B = 1000) with 95% CI. Calibration plots for 3- and 5-year survival are presented in Supplementary Figure S1

Among the 23 overlapping differentially expressed MTRGs, seven genes were associated with prognosis of glioma (Fig. 4b). When the penalty parameter lambda was the minimum, there were seven genes meeting the conditions of constructing the signature *ACSL1* (Acyl-CoA Synthetase Long Chain Family Member 1), *NOX4* (NADPH Oxidase 4), *ALDH3A2* (Aldehyde Dehydrogenase 3 Family Member A2), *ALKBH4* (AlkB Homolog 4), *FASTK* (Fas Activated Serine/Threonine Kinase), *FDX1* (Ferredoxin 1) et *ACADVL* (Acyl-CoA Dehydrogenase Very Long Chain) (Fig. 4b). The signature was formulated as (2.01E-04 ∗ ACSL1) + (-1.73E-03 ∗ NOX4) + (-3.92E-05 ∗ ALDH3A2) + (3.26E-03 ∗ ALKBH4) + (3.75E-05 ∗ FASTK) + (-2.82E-04 ∗ FDX1) + (1.88E-04 ∗ ACADVL). Positive coefficients suggest that increased gene expression is associated with a higher risk, while negative coefficients reflect a protective effect. Accordingly, ACSL1, ALKBH4, FASTK, and ACADVL are associated with higher risk, whereas NOX4, ALDH3A2, and FDX1 exert a protective effect on glioma prognosis. Survival analysis of the TCGA cohort demonstrated that patients in the H-R group had a significantly poorer prognosis than those in the L-R group (Fig. 5a). Cox regression analyses showed that the risk score served as an independent predictor of glioma prognosis (HR = 2.17, 95% CI: 1.44–3.30, *p* < 0.001) (Fig. 5b). VIF analysis indicated no significant multicollinearity between the risk score and clinical variables in the TCGA cohort (VIF = 1.53), supporting the stability of the multivariate model. Moreover, time-dependent ROC curve analysis demonstrated the predictive performance of the risk score, with area under the curve (AUC) values of 0.855, 0.874, and 0.815 at 1-, 3-, and 5-year survival, respectively (Fig. 5c). Consistently, the model achieved a C-index of 0.815 (Fig. 5a). Calibration curves showed good agreement between predicted and observed survival probabilities at 1-year OS in the TCGA cohort, indicating satisfactory model calibration (Fig. 5d).

### Validation of prognostic model built from MRGS

**Fig. 6:**
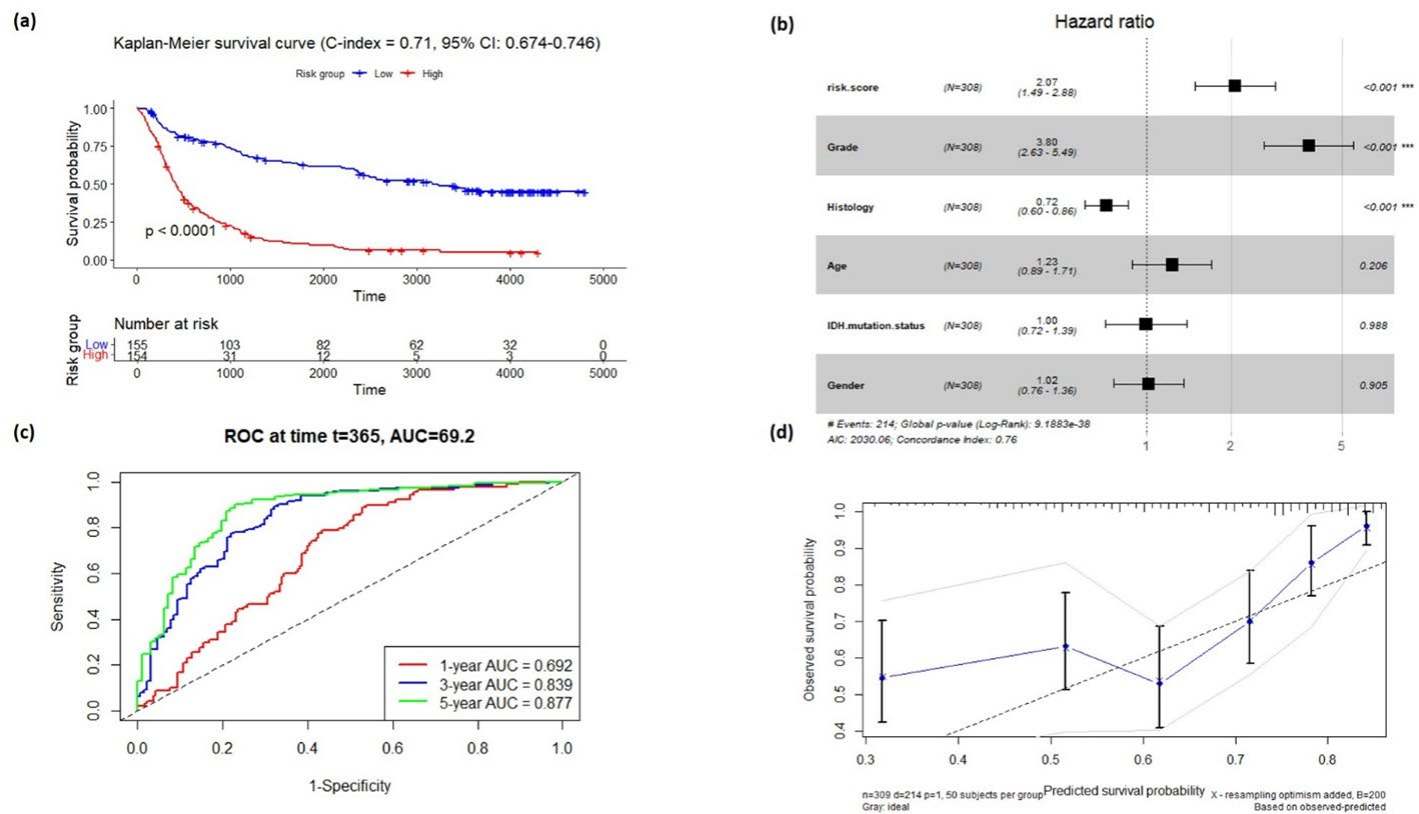
Performance of the prognostic signature in the CGGA cohort. **a** Kaplan–Meier survival curves comparing H-R and L-R groups based on the median cutoff. Blue curve represents the L-R group, and red curve represents the H-R group. “n” indicates the number of patients in each group. **b** Multivariate Cox regression analysis of prognostic factors. The risk score was included as a covariate. A HR greater than 1 indicates that higher risk score is associated with poorer prognosis. (full statistical details in Supplementary Table S4) ). **c** Time-dependent ROC curves at 1-, 3-, and 5-year for OS. **d** 1-year calibration plot demonstrating agreement between predicted and observed survival probabilities. The dashed line represents perfect calibration, while the blue curve corresponds to the bootstrap-corrected estimates (B = 1000) with 95% CI. Calibration plots for 3- and 5-year survival are presented in Supplementary Figure S1

To assess the robustness of the mitochondrial-related prognostic signature, the risk score model developed for the TCGA cohort was applied to the independent CGGA dataset (*n* = 325). According to Kaplan-Meier survival analysis, patients in the H-R group had significantly lower OS than those in the L-R group (*p* < 0.001) (Fig. 6a). After adjusting for clinical characteristics such as age, tumor grade, and IDH mutation status, multivariate Cox regression analysis revealed that the risk score was an independent predictor of OS in the CGGA cohort (HR = 2.07, 95% CI: 1.49–2.88, *p* < 0.001) (Fig. 6b). Consistent with the findings in the TCGA cohort, evaluation of multicollinearity in the CGGA validation set showed low VIF values for all included variables (VIF = 1.35), indicating no significant multicollinearity between the MRGS and clinical covariates. These results further support the stability and robustness of the multivariate Cox regression model in an independent cohort. To assess the incremental prognostic value of the risk score, hierarchical Cox regression analysis was performed. A baseline clinical model including tumor grade and IDH mutation status was first constructed, followed by the addition of the risk score. The inclusion of the risk score significantly improved model fit (χ² = 25.803, *p* = 3.78 × 10⁻⁷), indicating that it provides additional prognostic value beyond established clinical variables. Time-dependent ROC curve analysis further confirmed the predictive performance of the model, with area under the curve (AUC) values of 0.692, 0.839, and 0.877 for 1-, 3-, and 5-year survival, respectively (Fig. 6c). Consistently, the model achieved a C-index of 0.71 (Fig. 6a). Calibration curves of the nomogram in the CGGA cohort demonstrated good agreement between predicted and observed 1-year OS, indicating satisfactory calibration (Fig. 6d).

### Comparison of prognostic performance with previously reported models

**Fig. 7:**
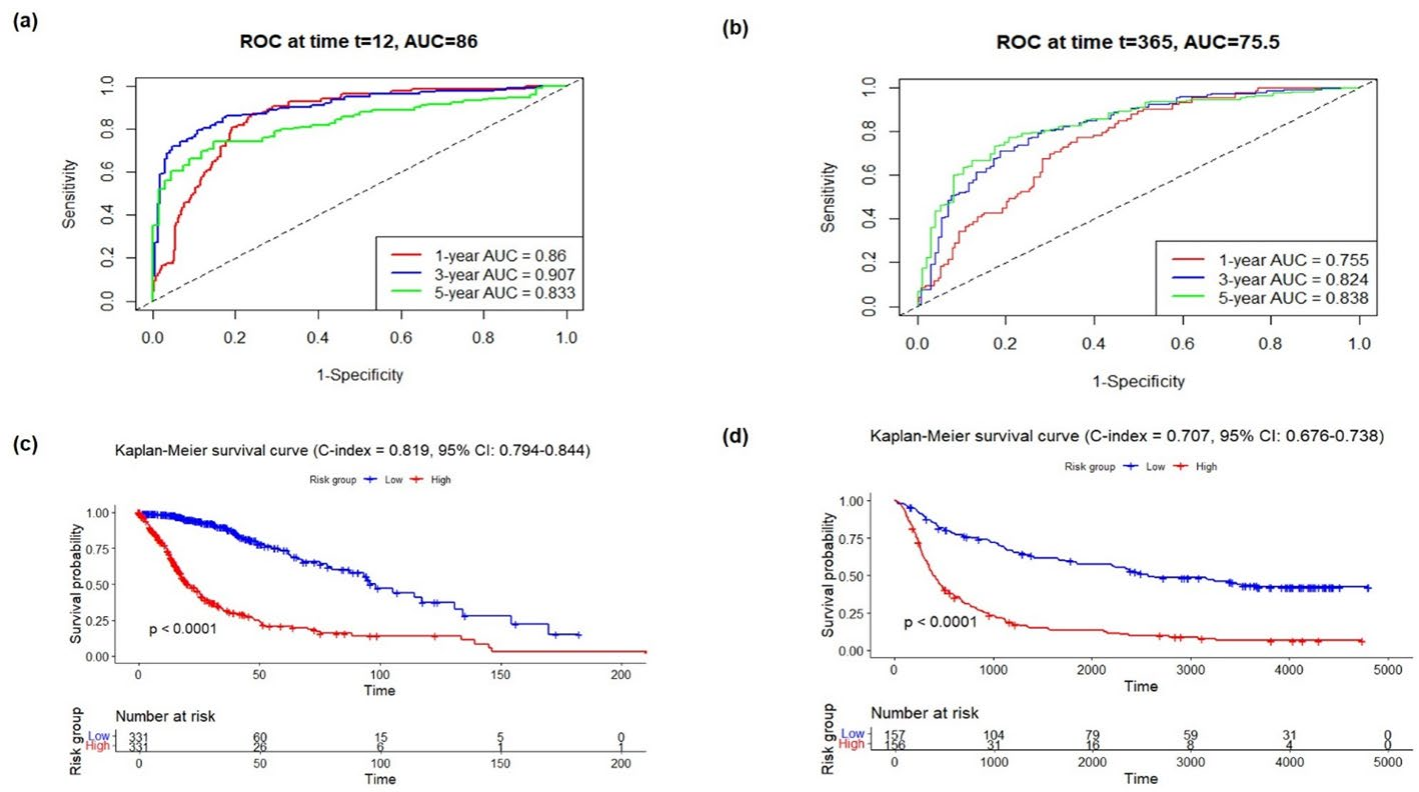
Prognostic performance of previously reported mitochondrial-related models. **a**,** b** Time-dependent ROC curves at 1-, 3-, and 5-year of OS for previously published models in the TCGA and CGGA cohort respectively. **c**,** d** C-index of the previously reported models in the TCGA and CGGA cohorts respectively, revealing their discriminative ability

We next compared the prognostic performance of our 7-gene mitochondrial signature with previously reported mitochondrial-related models [20]. As shown in Fig. 7, our model demonstrated comparable predictive performance, with high AUC values for 1-, 3-, and 5-year OS in both the TCGA (0.860, 0.907, and 0.833) and CGGA (0.755, 0.824, and 0.838) cohorts. Similarly, the C-index of our signature was largely comparable to those reported in previously published models, reaching 0.819 in the TCGA and 0.707 in the CGGA cohort.

### Association between risk score and clinicopathological characteristics

**Fig. 8:**
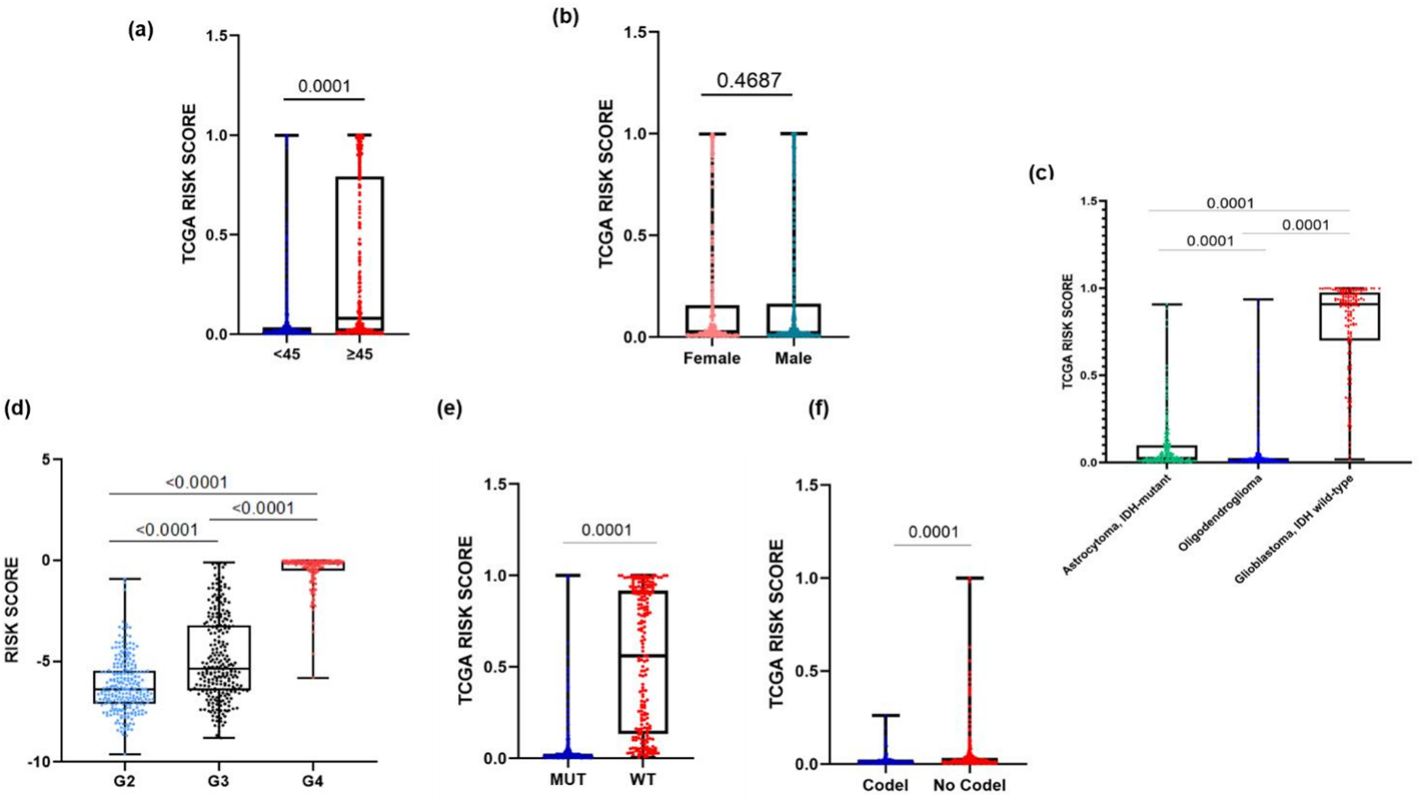
Association between the mitochondrial-related risk score and clinicopathological features in the TCGA cohort. **a** The risk score expression profile stratified by age (< 45 vs. ≥ 45 years). **b** The risk score expression profile according to sex. **c** Risk score across glioma histological subtypes. **d** Risk score according to WHO grade. **e** Risk score according to IDH mutation status (wild-type vs. mutant). **f** Risk score according to 1p/19q co-deletion status. Statistical comparisons were assessed using the Mann–Whitney test

**Fig. 9:**
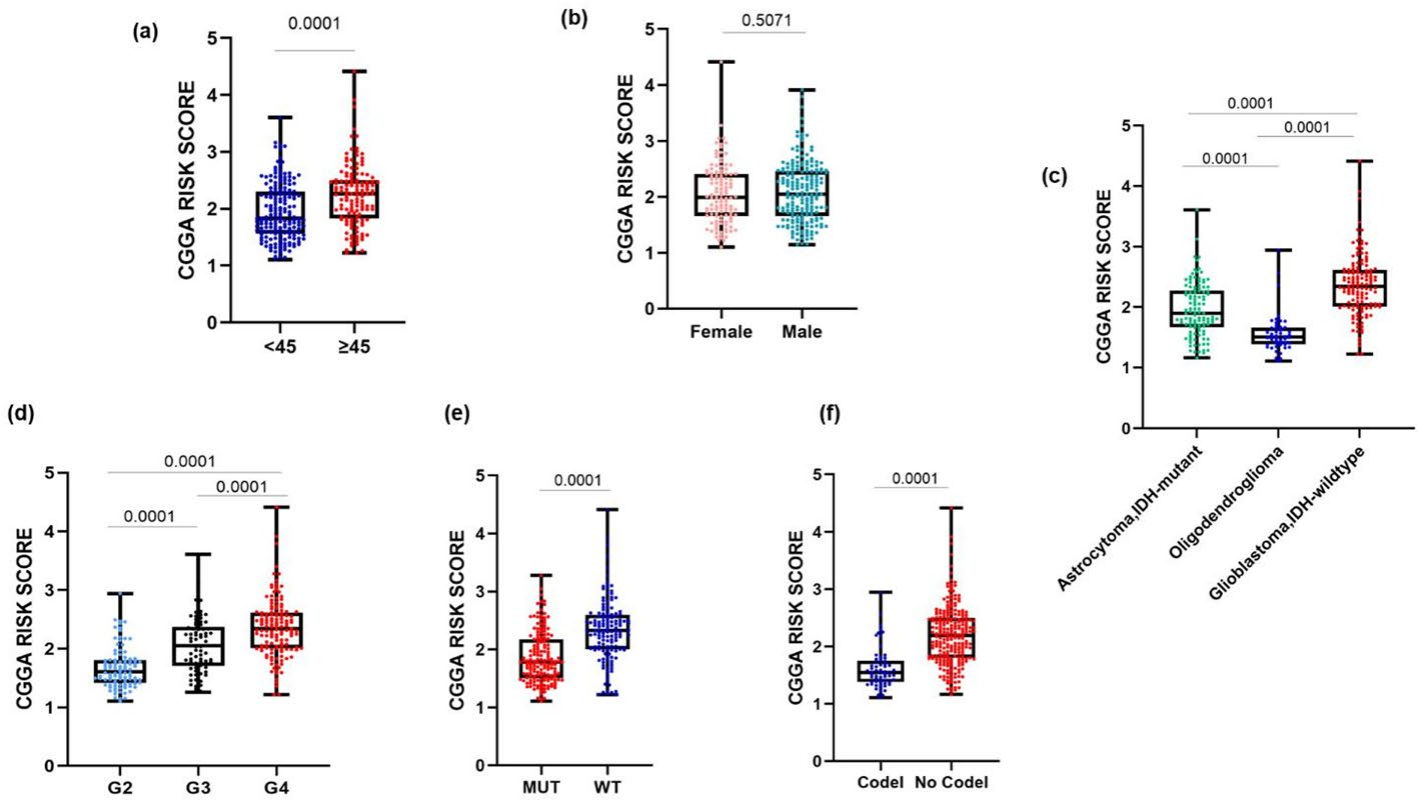
Association between the mitochondrial-related risk score and clinicopathological features in the CGGA cohort. **a** The risk score expression profile stratified by age (< 45 vs. ≥ 45 years). **b** The risk score expression profile according to sex. **c** Risk score across glioma histological subtypes. **d** Risk score according to WHO grade. **e** Risk score according to IDH mutation status (wild-type vs. mutant). **f** Risk score according to 1p/19q co-deletion status. Statistical comparisons were assessed using the Mann–Whitney test

Based on clinical data from the TCGA and CGGA cohorts, we investigated the association between the risk score and clinicopathological characteristics. Our results revealed a significant association between higher risk scores and higher tumor grade (*p* = 0.0001), histological subtype (*p* = 0.0001), 1p19q codeletion (*p* = 0.0001), Isocitrate Dehydrogenase (IDH) status (*p* = 0.0001) and age (*p* = 0.0001), all of which have been reported to correlate with glioma prognosis. In contrast, sex (*p* = 0.4687) was not significantly associated with the risk score (Fig. 8) and (Fig. 9).

### Independent prognostic performance across clinical subgroup

**Fig. 10:**
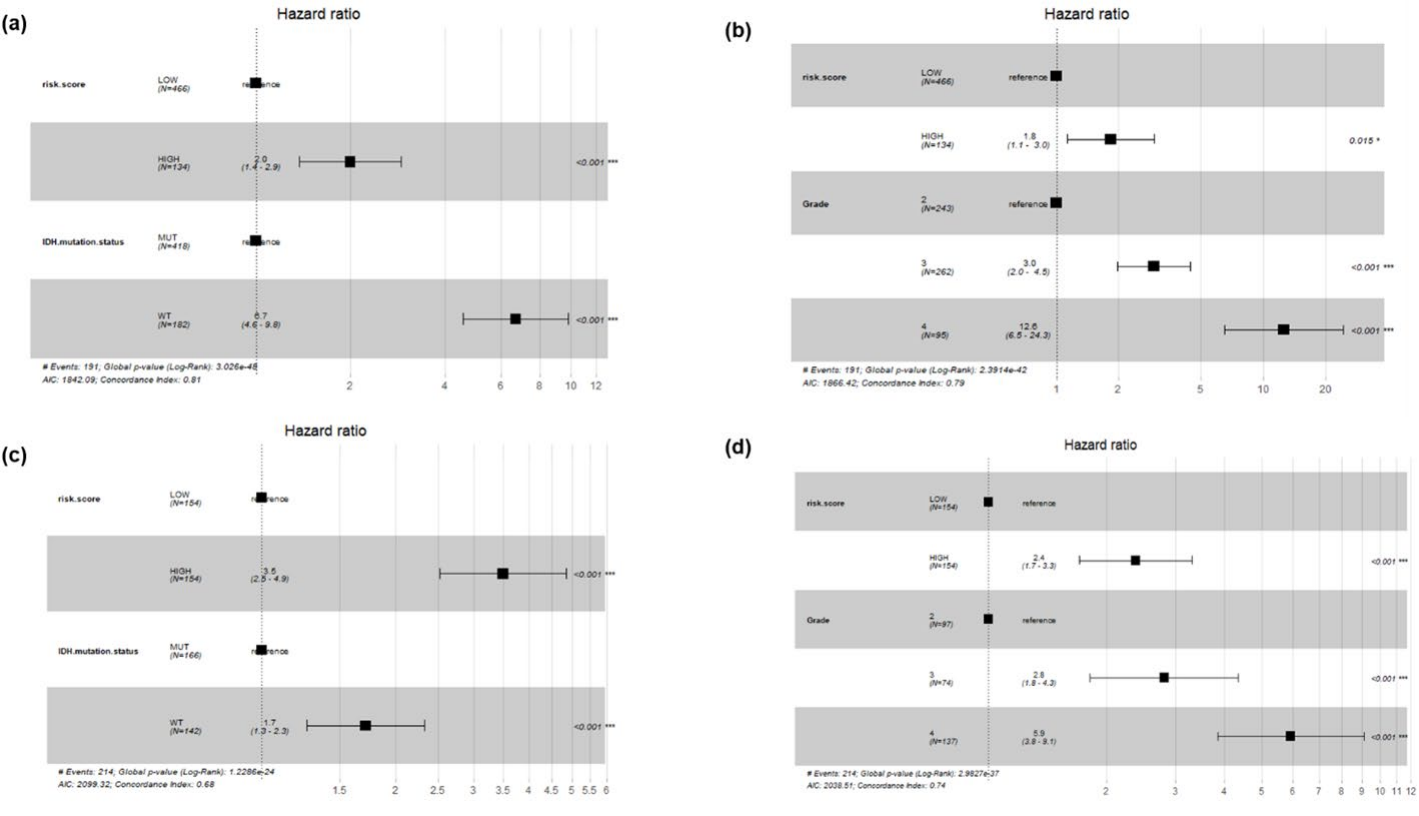
Univariate Cox regression analysis of the mitochondrial risk signature in clinically relevant subgroups.** a**,** b** TCGA cohort: association between the risk score and OS, stratified by IDH mutation status and WHO grade. **c**,** d** CGGA cohort: association between the risk score and OS, stratified by IDH mutation status and WHO grade. HR with 95% CI are shown

Subgroup analyses were conducted to evaluate the prognostic value of the MRGS within clinically relevant groups **(**Fig. 10**).** The risk score remained strongly associated with OS regardless of WHO grades (G2, G3, G4) and IDH status (wild-type and mutant), thereby demonstrating that our signature provides additional predictive information beyond established clinical markers.

### Association between mitochondrial-related risk score and immune signatures in glioma

**Fig. 11:**
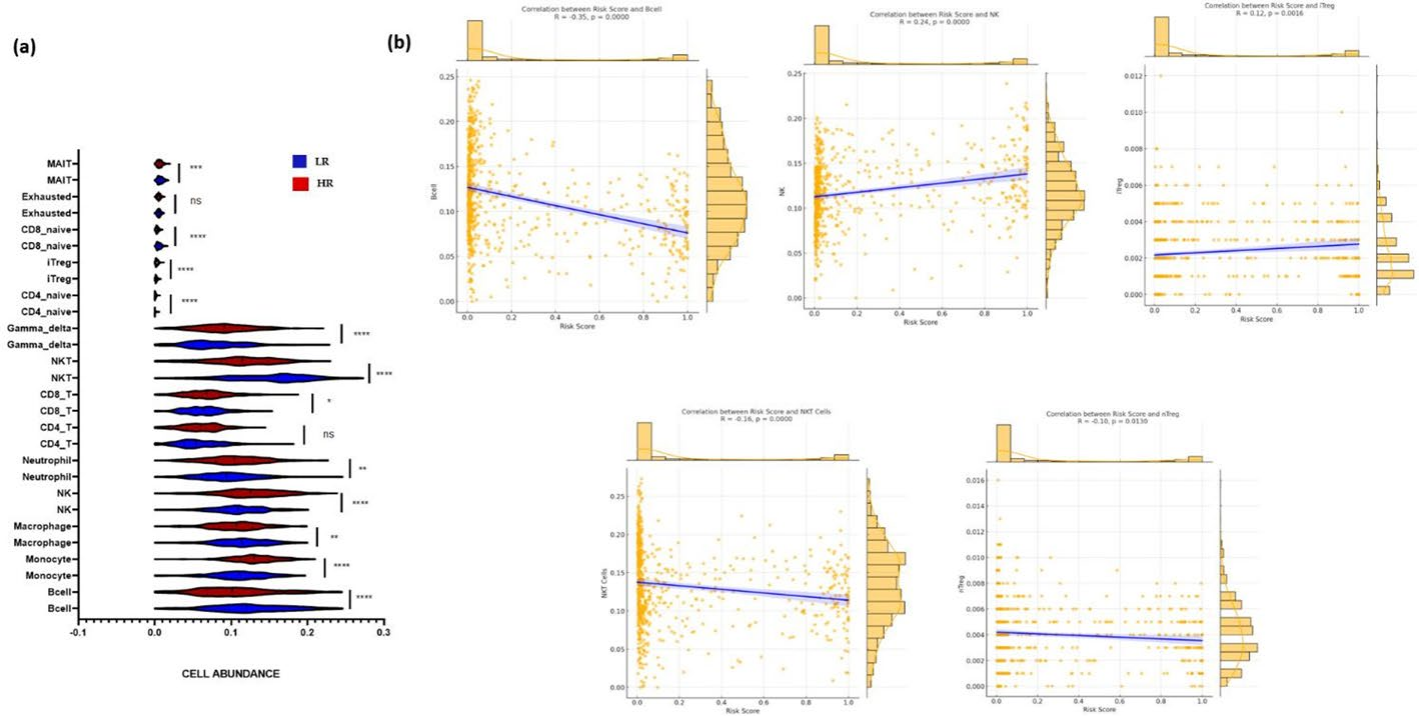
Association between the mitochondrial-related risk score and immune cell patterns in the TCGA cohort. **a** Differential distribution of immune cell fractions between L-R and H-R groups evaluated using the ImmuneCellAI algorithm. Group comparisons were performed using the Mann–Whitney test. **b** Correlation between the risk score and immune cell subsets (B cells, NK cells, NKT cells, nTreg, and iTreg based on Spearman correlation analysis. Statistical significance is indicated as follows: *p < 0.05, **p < 0.01, ***p < 0.001, ****p < 0.0001; ns, not significant

ImmuneCellAI analysis were assessed to analyze the differential distribution of immune cell fractions between both groups of patients (L-R and H-R) in TCGA cohort. We found that the H-R group had significantly higher levels of induced regulatory T cells (iTregs), gamma-delta T cells, CD8 + T cells, CD4 + T cells, NK cells, mucosa-associated invariant T cells (MAITs), neutrophils, and monocytes. In contrast, naïve CD8 + T cells, NKT cells, macrophages, and B cells were more abundant in the L-R group (Fig. 11a). Moreover, correlation analysis showed that the risk score was negatively correlated with B cells, NKT cells, and natural T regulatory cells (nTregs), while positively correlated with the infiltration of iTregs and NK cells (Fig. 11b**).** Notably, CD8 + T cell infiltration did not show a significant correlation with the risk score.

**Fig. 12:**
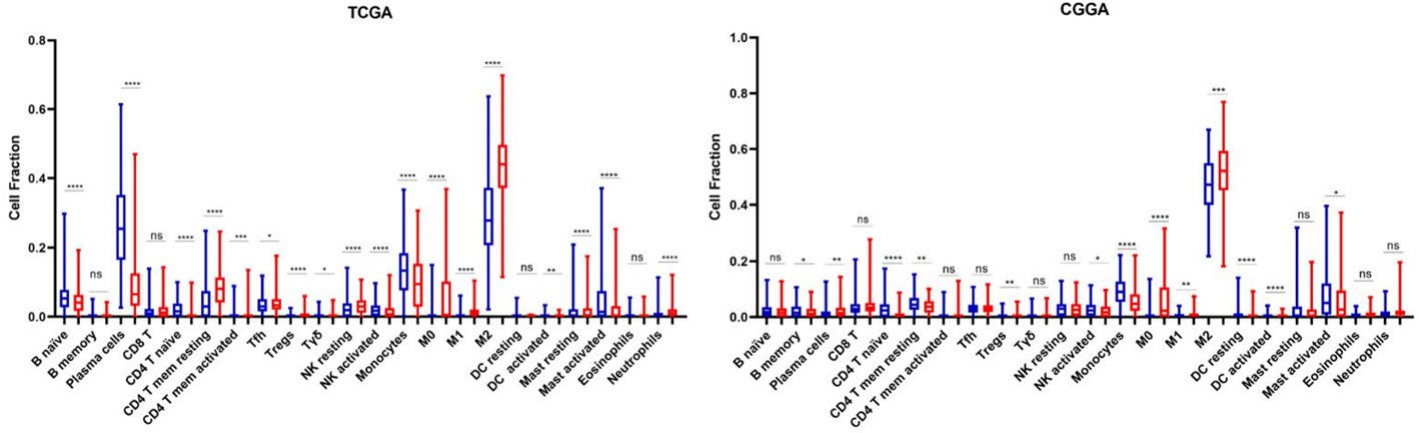
Immune cell infiltration analysis based on the CIBERSORT algorithm in TCGA and CGGA cohorts. Differential distribution of immune cell fractions between H-R and L-R groups using the CIBERSORT algorithm. Differences in immune cell fractions between H-R and L-R groups were assessed using Mann-Whitney Test. Statistical significance is indicated as follows: *p < 0.05, **p < 0.01, ***p < 0.001, ****p < 0.0001; ns, not significant

To further validate the immune landscape linked to the MRGS, immune cell infiltration was also analyzed using the CIBERSORT algorithm in both TCGA and CGGA cohorts (Fig. 12). In the TCGA cohort, the H-R group display a significantly higher infiltration of immunosuppressive cell types, such as regulatory T cells (Tregs), M2 macrophages, resting NK cells, and neutrophils. On the other hand, several important immune populations such as activated NK cells, naïve CD4 + T cells, naïve B cells, plasma cells, and activated dendritic cells were decreased in the same group. Notably, no statistically significant difference in CD8 + T cell infiltration was observed between the H-R and L-R groups. Similar immune patterns were observed in the CGGA cohort, where the H-R group also displayed higher levels of immunosuppressive cells (Tregs and M2 macrophages) and lower levels of naïve CD4 + T cells and activated NK cells. Similarly, CD8 + T-cell infiltration showed no significant differences between the two risk groups. Overall, these data suggest that the H-R group shows an immune-related transcriptional pattern consistent with an immunosuppressive TME. However, these findings are correlative and require further experimental validation.

**Fig. 13:**
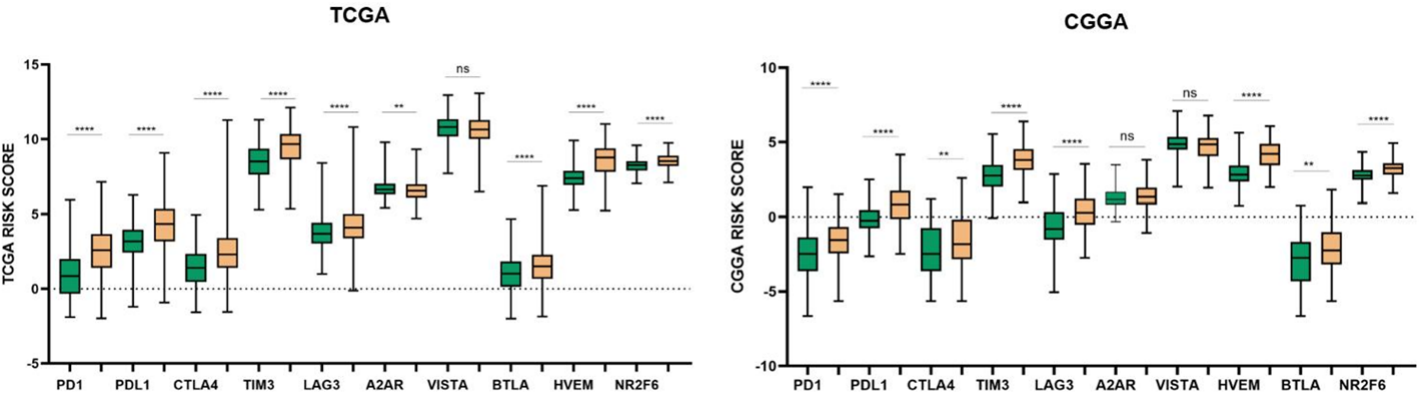
Correlation between the risk score and inhibitory immune checkpoints in glioma in TCGA and CGGA cohorts. The MRGS was analyzed for its association with inhibitory immune checkpoint molecules in TME of glioma. Correlation analyses were performed in both TCGA and CGGA cohorts. A positive correlation was observed between the MRGS and the expression levels of multiple immune checkpoints, including PD-1, PD-L1, CTLA-4, LAG-3, TIM-3, BTLA, HVEM, and NR2F6. Correlations were assessed using Spearman correlation analysis. Differences between groups were assessed using Mann-Whitney Test. Statistical significance is indicated as follows: *p < 0.05, **p < 0.01, ***p < 0.001, ****p < 0.0001; ns, not significant

We next explored the relationship between the mitochondrial-related risk score and the expression of immune checkpoint markers in the glioma TME. Our data revealed that several well-known immune checkpoint genes, including PD-1, PD-L1, CTLA-4, LAG-3, TIM-3, BTLA, HVEM and NR2F6, were considerably upregulated in the H-R group compared to the L-R group. PD-1 (*p* < 0.0001), PD-L1 (*p* < 0.0001), CTLA-4 (*p* < 0.0001), LAG3 (*p* < 0.0001), TIM3 (*p* < 0.0001), BTLA (*p* < 0.0001), HVEM (*p* < 0.0001) and NR2F6 (*p* < 0.0001) in TCGA and CGGA cohorts (Fig. 13). These findings demonstrated an association between the risk score and ICP expression patterns in glioma.

### Functional enrichment analysis of the MRGS

**Fig. 14:**
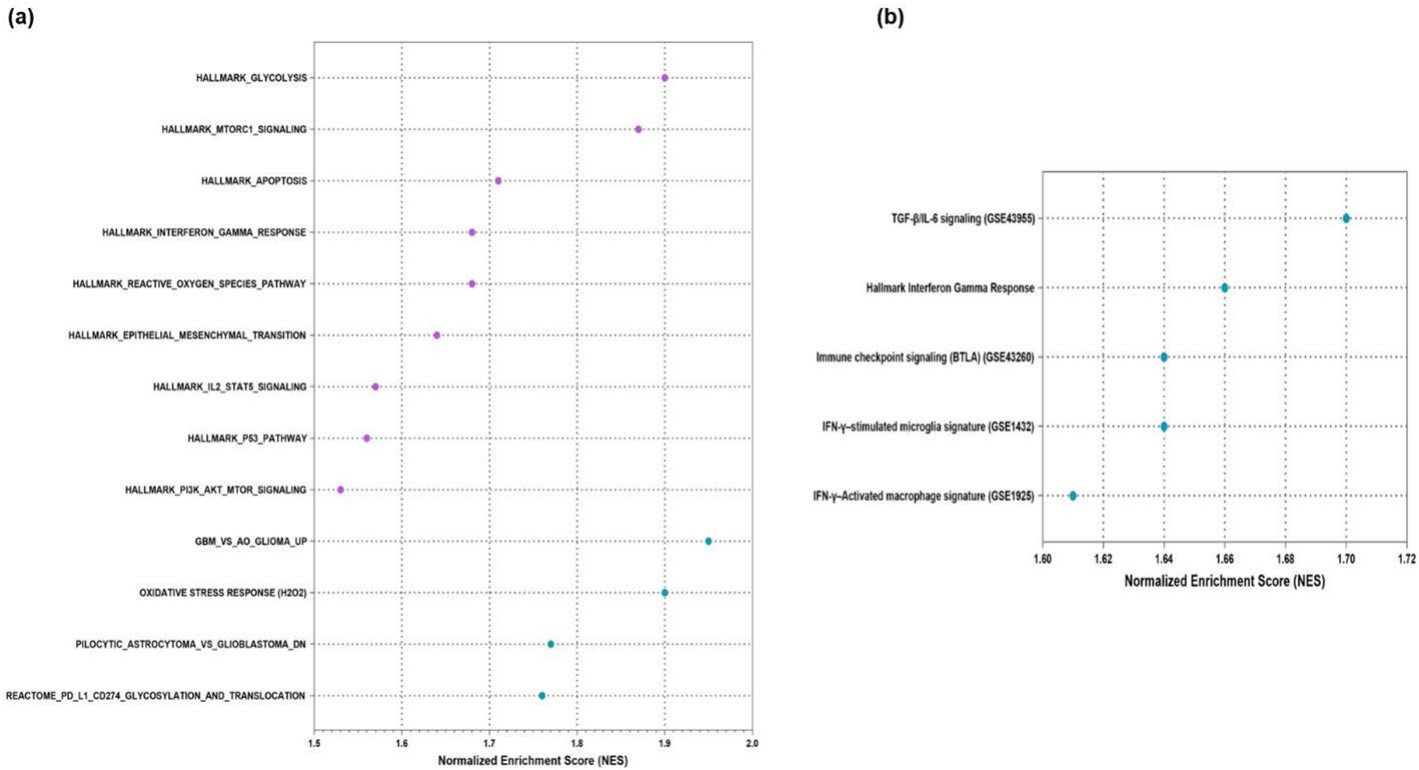
Functional enrichment analysis of our 7-gene signature-derived risk score. Dot plot showing GSEA (biological process, cellular process and molecular and immunological function) for H-R score expression based on the **a** TCGA and **b** CGGA cohorts. Gene sets with |NES| > 1, nominal p-value < 0.05, and FDR < 0.25 were considered significantly enriched

Finally, to better understand the biological mechanisms related to H-R group expression, we conducted enrichment analyses of metabolic pathways and molecular signatures between the H-R and L-R groups defined by the 7-gene signature-derived risk score. Our analysis showed that the H-R group exhibited significant enrichment in pathways related to tumor progression, metabolic reprogramming, and immune-related processes. More specifically, we noted enrichment in the epithelial-mesenchymal transition (EMT), the PI3K-AKT-mTOR signaling pathway, the mTORC1 signaling pathway, and glycolysis. Furthermore, pathways related to oxidative stress, notably ROS signaling and the H₂O₂-induced oxidative stress response, were highly enriched. Immune pathways, such as PD-L1 signaling and IL2-STAT5 signaling, were also enriched, indicating alterations in the TIME. Moreover, genetic signatures associated with GBM and tumor grade progression (GBM vs. lower-grade glioma) were enriched in the H-R group, confirming the association between the risk score and more aggressive disease subtypes (Fig. 14a). In the CGGA cohort, GSEA analysis consistently supported the enrichment of immune pathways in the H-R group, specifically those associated with the interferon-gamma response, macrophage/microglia activation, immune checkpoint signaling (BTLA), and TGF-β/IL-6 signaling (Fig. 14b).

## Discussion

Various prognostic models based on MTRGs have been uncovered for various types of cancers, including bladder and prostate cancer [21, 22]. Yet a predictive model based on MTRGs for glioma patient has rarely been reported. Our findings contribute to the growing body of research integrating computational and multi-omics approaches in glioma research, underscoring the translational potential of MTRGS in immune microenvironment regulation and glioma prognosis [14, 15].

In the present investigation, we developed a mitochondrial-related 7-gene signature, ACSL1, NOX4, ALDH3A2, ALKBH4, FASTK, FDX1, and ACADVL, evaluated its prognostic value in the TCGA cohort (Fig. 5), and validated it in an independent CGGA cohort (Fig. 6). Although the coefficients in the final LASSO model were numerically small, this can be attributed to the normalization and scaling of the gene expression data. Importantly, the risk score derived from these coefficients remains clinically interpretable, as it indicates the relative contribution of each gene to patient prognosis. Notably, NOX4 exhibits a negative coefficient in the LASSO model despite its H-R role in univariate analysis, which may reflect multicollinearity or compensatory interactions among the signature genes. Identified genes have been previously reported as implicated in tumor progression and metabolic reprogramming (Supplementary Table. S5). For example, ACSL1 and ACADVL are involved in lipid metabolism and energy production, and have been associated with poor prognosis in several cancers, including breast and colorectal cancer [32, 33]. In low grade glioma, ACSL1 provides valuable insights into prognosis and therapeutic targeting as it regulates immune cell infiltration and anti-tumor immune responses [23]. NOX4, a generator of ROS, promotes tumor angiogenesis and invasion, and modulates VEGF, PI3K/AKT, and TGF-β signaling pathways which are relevant in immune regulation within the TME [24]. The role of NOX4 in GBM has been linked to cell growth, survival, invasion, and therapeutic resistance through hypoxia-induced radiation [25]. ALDH3A2, a key player of long-chain aliphatic aldehydes metabolism, shows a significant upregulated expression at the protein level in cancer samples [26, 27]. ALKBH4 has been described as a tumor suppressor with potential therapeutic relevance in hypoxic tumors [28]. However, studies have also reported its upregulation in gastric cancer tissues compared to adjacent normal tissues, where it correlates with poor prognosis, suggesting a context-dependent role in tumor progression [29]. FASTK regulates mitochondrial RNA processing, influencing mitochondrial gene expression and bioenergetics and showed strong overexpression in highly malignant pancreatic ductal adenocarcinoma and various types of cystic pancreatic tumors, with potential for malignant transformation [30]. FDX1 contributes to electron transport and redox reactions, affecting ROS homeostasis, thereby involved in tumor initiation and progression [31]. Silencing of FDX1 inhibits glioma cell invasion and migration, which is accompanied by inactivation of the NOD-like receptor signaling pathway, suggesting that FDX1 can promote tumor aggressiveness through inflammatory pathways [32]. Collectively, dysregulation of these genes may contribute to glioma progression through coordinated mitochondrial function alterations, including oxidative phosphorylation and energy homeostasis, leading to enhanced metabolic flexibility of tumor cells. Such mitochondrial dysfunction is often associated with increased production of ROS, which can promote genomic instability and activate pro-tumorigenic pathways [33].In parallel, metabolic reprogramming may facilitate adaptation to the hypoxic TME, support invasive phenotypes, and indirectly modulate the TIME by influencing immune cell infiltration and functions [34, 35]. While certain genes in our model have been previously associated with glioma prognosis, the strength of our strategy lies in integrating them into a multigene signature that accounts for distinct yet complementary mitochondrial processes. This combinatorial approach could improve prognostic performance and more accurately reflect the complex biological interactions underlying glioma progression.

The TCGA survival analysis showed that patients in the H-R group had a significantly worse prognosis than those in the L-R group (Fig. 5a). Similar results were observed in the CGGA cohort, confirming the reproducibility of the signature over independent datasets (Fig. 6a). Although the median cut-off was used for risk stratification in this study, it is a relatively arbitrary cut-off. Further research should consider more reliable approaches, such as maximum selected rank statistics, thresholds based on the ROC curve, or thresholds based on clinical data, in order to improve prognostic discrimination and facilitate clinical translation.

Tumor grade, age, histology and molecular subtype remain among the most frequently used factors for prognostic prediction and treatment strategies in glioma [36]. Our model effectively stratified patients based on survival, with H-R groups showing poorer outcomes than L-R groups. Multivariate Cox regression analysis revealed that the MRGS remained significantly linked to OS after adjustment for clinical variables in both the TCGA and CGGA cohorts. However, its interpretation as a completely independent prognostic factor should be interpreted with caution. In particular, the lack of statistical significance of established markers such as IDH status in the CGGA cohort may reflect cohort-specific characteristics, variable interactions, or sample distribution differences. Additionally, VIF analysis revealed no multicollinearity in either the training (TCGA) or validation (CGGA) cohorts, suggesting a lack of significant multicollinearity among the variables included in the model. Furthermore, hierarchical Cox regression analysis showed that including the risk score significantly improved model performance, suggesting that it provides additional prognostic value beyond established clinical parameters. Collectively, these results support the potential complementary value of the MRGS while highlighting the need for cautious interpretation and further validation.

In parallel, the MRGS demonstrated strong predictive performance, as evidenced by high AUC values and C-indexes in both the TCGA and CGGA cohorts (Figs. 5 and 6). However, a lower C-index was observed in the CGGA compared to TCGA cohorts (0.71 vs. 0.815), indicating a decline in the model’s performance during external validation. This reduction suggests potential cohort-dependent variability and the model’s potential sensitivity to dataset-specific characteristics. This may be due to population heterogeneity, differences in sequencing platforms, batch effects, and variations in clinical characteristics between cohorts. Furthermore, variability in the direction and magnitude of gene-level effects across datasets may indicate underlying biological heterogeneity, limited robustness, and potential instability of the model across independent cohorts. Although the model retained moderate discriminatory performance in the external validation cohort, these findings raise concerns about its generalizability across heterogeneous patient populations and technical settings. These results underscore the need for further validation on larger independent cohorts before clinical application.

Calibration performance showed good agreement at 1 year in both the TCGA and CGGA cohorts, supporting the model’s reliability for short-term survival prediction (Figs. 5d and 6d). At longer follow-up times (3 and 5 years), calibration accuracy decreased, with a consistent tendency to overestimate survival probabilities, particularly in low- and intermediate-risk ranges, observed in both datasets. This likely reflects increased biological heterogeneity, differences in therapeutic strategies and patient characteristics, besides the impact of censoring over extended follow-up periods. Importantly, these deviations indicate that, despite the model’s utility for population-level risk stratification, its reliability for accurate clinical prediction at the individual level remains limited, particularly for long-term survival estimation. Furthermore, quantitative calibration metrics such as calibration slope, intercept, and Brier score were not evaluated in the present study, which constitutes an additional limitation. Future studies integrating larger multicenter cohorts, external prospective validation, and recalibration strategies should be conducted to improve long-term predictive accuracy and clinical applicability.

Moreover, we analyzed the expression profile of the risk score as well as its biological and clinical associations in glioma. The risk score expression was significantly higher in aggressive glioma grades (G3 and G4) and pathological subtypes particularly GBMs. Furthermore, the high risk score was significantly enriched in well-known malignant molecule phenotype; the IDH wild-type state, along with the 1p19q co-deletion (Fig. 8). All of these observations suggest that the risk score could act as a potent biomarker for GBM diagnosis and grading. Similar associations were observed in the CGGA cohort, supporting the model’s robustness and generalizability (Fig. 9).

In addition, the risk score was strongly associated with OS, regardless of tumor grades, and across IDH mutation subgroups (Fig. 9). These findings suggest that this signature provides prognostic information beyond established clinical biomarkers and is capable of identifying H-R patients, even within clinically homogeneous groups. This added value highlights the potential utility of the mitochondrial signature for risk stratification and personalized treatment of glioma patients. By combining mitochondrial gene expression with characteristics of the TIME, the current signature may provide complementary information that reflects aspects of tumor heterogeneity not captured by conventional markers. Future research, including direct comparisons using measures such as net reclassification improvement (NRI) or integrated discrimination improvement (IDI), is needed to formally evaluate the additional predictive value and clinical relevance of this gene profile.

ICPs modulate antitumor immunity and their overexpression has frequently been associated with poor prognosis in several cancers [37]. In our study, we observed a positive correlation between the mitochondrial-related risk score and the expression of multiple ICPs in the glioma TME including PD-1, PD-L1, CTLA-4, LAG-3, TIM3, BTLA, NR2F6, and HVEM in both TCGA and CGGA cohort highlighting the external validity of the signature (Fig. 13). Research have indicated that overexpression of ICPs such as PD-1, LAG-3, VISTA, B7-H3 and TIM-3 in glioma are associated with T cell dysfunction and facilitates tumor immune evasion [38–42]. These findings suggest that the H-R group is associated with a deeply immunosuppressive TME, which may be associated with the observed poorer prognosis. Importantly, these correlations may be influenced by tumor grade and IDH status, which are known to affect both ICPs expression and immune infiltration. Consequently, these observations are correlational in nature and do not imply a direct causal or mechanistic relationship; they therefore require further experimental validation.

GSEA enrichment analyses reveal insights into the biological mechanisms underlying the H-R group defined by our 7- MRGS. The H-R tumors show an activation of tumor progression, metabolic reprogramming, and immune dysregulation pathways. The enrichment of the EMT, PI3K–AKT–mTOR, and mTORC1 signaling pathways, as well as glycolysis, suggests heightened proliferative and invasive potential, along with metabolic adaptation for rapid tumor growth. Concurrently, the H-R group showed activation of oxidative stress pathways, notably ROS signaling and the H₂O₂-induced oxidative stress response, which corresponds to mitochondrial dysfunction and increased ROS production likely to further facilitate tumor growth. Immune-related pathways were also significantly enriched in H-R tumors. Associated signaling pathways including PD-L1, IL-2–STAT5, IFN-γ responses, TGF-β/IL-6-mediated Th17 signaling, BTLA, suggest the existence of a complex immunosuppressive TME, contributing to immune evasion and poor clinical outcomes. Collectively, in H-R gliomas, MRGS represents a convergent pattern of mitochondrial dysfunction, metabolic reprogramming, proliferative and invasive potential, and immunosuppressive microenvironment. However, while our findings are based on transcriptomic correlations, these results generate verifiable hypotheses for future studies, particularly the functional validation of mitochondrial pathways and immune interactions as potential combinatory therapeutic approches.

Cancer immunotherapy efficacy is dependent on a thorough understanding of the TME, in which the tumor, stroma, and infiltrating immune cells interact in a complex network [43]. Previous studies have shown that immune cell signature within the TME may indicate the prognosis of several cancers [44–46]. In GBM, it has been shown that immune infiltration levels have a significant impact on the prognosis and tumor aggressiveness [47–49]. In our study, ImmuneCellAI with TCGA data, showed that Gamma Delta T cells, CD8 + T cells, NK cells, neutrophils, monocytes, MAIT cells, and iTregs were significantly enriched in the H-R group, whereas B cells, macrophages, NKT cells, and naïve CD8 + T cells were enriched in the L-R group. The CIBERSORT analysis showed that immunosuppressive populations, including Tregs, M2 macrophages, resting NK cells, and neutrophils, were more abundant in the H-R group. Conversely, immune cells associated with favorable prognosis, such as activated NK cells, naïve CD4 + T cells [50], M1 macrophages, and naïve B cells, were less abundant. Similarly, in the CGGA cohort, CIBERSORT revealed increased infiltration of immunosuppressive cells, Tregs and M2 macrophages, in the H-R group, as well as a decrease in the abundance of naïve CD4 + T cells, M1 macrophage, and activated NK cells. These results align with the correlation analysis demonstrating a positive correlation between the risk score and iTregs, as well as a negative correlation with B cells and NKT cells. However, no significant correlation was observed between CD8^+^ T cells and the risk score. Overall, these results consistently confirm that the H-R group is characterized by an immunosuppressive TME in both cohorts, favoring a poorer prognosis. However, these observations are based on transcriptomic deconvolution and require further validation using functional exhaustion markers and experimental approaches.

Recently, several mitochondrial-related prognostic markers have been identified in glioma [20]. Yang et al. developed a MRGS for predicting prognosis and therapeutic response in low-grade gliomas, while Liu et al. proposed a model based on mitochondrial genes associated with prognosis and immune characteristics in glioma patients. Differences in datasets, bioinformatics pipelines, and gene selection criteria most likely contributed to the identification of distinct gene signatures across studies. To further clarify the added value of our model, we performed a comparative analysis with previously published MRGS using the same TCGA and CGGA cohorts. Our results showed that the 7-gene signature exhibited comparable prognostic performance in terms of AUC and C-index. Although these results do not demonstrate clear superiority over existing models, they support the reproducibility of mitochondrial-based prognostic approaches across independent datasets. Importantly, our study extends previous work by providing an integrative framework linking mitochondrial-related gene expression with TIME characteristics. This combined analysis offers additional biological insight into the interplay between mitochondrial alterations and immune regulation in glioma. Moreover, validation across two independent cohorts and consistent performance observed in subgroup analyses suggest that the MRGS could provide complementary prognostic information within specific clinical contexts.

Overall, our results support the hypothesis that dysregulation of MTRGs promotes glioma progression through modulation of tumor aggressiveness and the immune microenvironment. We propose that mitochondrial gene alterations could influence tumor growth and immune evasion, contributing to glioma progression and patient prognosis. This integrated model provides a framework for understanding the interaction between mitochondrial function and the TIME in glioma, and may offer insights for future experimental validation and therapeutic targeting.

From a clinical perspective, the proposed signature could provide additional and complementary prognostic value beyond established clinical parameters. It could help identify patients who may require closer monitoring and more intensive treatment, while those at lower risk could benefit from standard management. It could also facilitate patient selection for clinical trials. However, its clinical use remains limited at this stage and requires further prospective validation.

Despite the promising results of this study, several limitations should be addressed. First, it relied on retrospective transcriptomic data from publicly available databases, which may contribute to selection bias and heterogeneity among patient samples. Second, although we created and validated the MRGS using independent datasets, prospective external validation in large multicenter cohorts remains necessary before any clinical application. In addition, the lack of calibration slope/intercept estimation, Brier score assessment, and decision curve analysis representes a notable limitation that should be addressed in future validation studies. Third, this study is based on in silico analyses, and experimental validation at the mRNA and protein levels would be necessary to validate the reliability of the results. Furthermore, additional mechanistic studies are required to clarify the biological links between the identified genes, mitochondrial dysfunction, and alterations in the TIME. Importantly, future research should explore how mitochondrial metabolic reprogramming and oxidative stress may influence immune cell function and tumor progression in glioma. Therefore, the current findings should be interpreted as hypothesis-generating rather than providing direct mechanistic or clinical conclusions. Finally, the use of bulk RNA-sequencing data may not fully capture intratumoral heterogeneity and complex cellular interactions. Advanced approaches such as single-cell or spatial transcriptomics could provide deeper insights into the interaction between mitochondrial pathways and the immune microenvironment.

Finally, in recent years, technological innovations, such as the integration of Internet of Things (IoT) systems into medical practice, have dramatically improved data collection, remote monitoring, and personalized medical care. In this context, digital health approaches, including Internet of Things (IoT)-based systems, may facilitate continuous patient monitoring and real-time integration of clinical and molecular data, potentially complementing prognostic models such as the mitochondrial-related gene signature (MRGS) proposed in this study [51]. Furthermore, advanced imaging modalities, including radiotherapy planning based on 18 F-FDG PET-CT, have shown promise in improving target delineation and treatment accuracy in patients with glioma. The integration of molecular signatures such as the MRGS with metabolic and imaging-based biomarkers may further enhance risk stratification and support more individualized therapeutic decision-making [52, 53]. In addition, 3D printing technologies have been increasingly used in neurosurgical practice, education, and patient-specific modeling. Such advances underscore the growing importance of precision medicine in glioma treatment, where genomic profiling including mitochondrial dysfunction–based signatures, can be integrated with technological advances to improve clinical outcomes [54].

## Conclusion

In the present study, we developed and validated a mitochondrial-related seven-gene signature with substantial predictive value in glioma patients. This risk model successfully stratifies patients into H-R and L-R groups based on overall survival rates and aggressive clinical and immunological features. Compared with previously reported mitochondrial signatures, our model was validated across independent cohorts and linked to both molecular and immune characteristics, highlighting its potential utility for prognostic stratification and therapeutic guidance. These findings shed new light on mitochondrial dysfunction in glioma and suggest potential markers for prognosis and therapeutic targeting. However, further prospective and experimental studies are needed before clinical application.

## Supplementary Information

Below is the link to the electronic supplementary material.

Supplementary Material 1

Supplementary Material 2

Supplementary Material 3

Supplementary Material 4

Supplementary Material 5

Supplementary Material 6

## Data Availability

The datasets generated and/or analysed during the current study were obtained from publicly accessible databases. The TCGA LGG (lgg_tcga_pancancer) and TCGA GBM (gbm_tcga_pan_cancer) cohorts are freely available through the cBioPortal platform ( [https://www.cbioportal.org/](https:/www.cbioportal.org) ). The CGGA cohort, consisting of 325 glioma tissue samples from a Chinese population, was downloaded from the CGGA database ( [https://www.cgga.org.cn/](https:/www.cgga.org.cn) ).

## Abbreviations

CGGA: Chinese Glioma Genome Atlas
CI: Confidence interval
CNS: Central nervous system
DEGs: Differential expressed gene
EMT: Epithelial mesenchymal transition
FDR: False discovery rate
GBM: Glioblastoma
HR: Hazard ratio
H-R: High-risk
IDH: Isocitrate dehydrogenase
IoT: Internet of Things
iTreg: Induced Treg
LASSO: Least Absolute Shrinkage and Selection Operator
L-R: Low-risk
MAIT: Mucosal associated invariant T cells
MRGS: Mitochondrial-related genes signature
MTRGs: Mitochondrial-related genes
nTreg: Natural Tregs
OS: Overall survival
TCGA: Cancer Genome Atlas
TILs: Tumor-infiltrating lymphocytes
TME: Tumor microenvironment
TIME: Tumor immune microenvironment
ROS: Reactive oxygen species
VIF: Variance inflation factor
