# Supplementary material for: A novel mitochondrial-related gene signature for assessing the tumor immune microenvironment and predicting prognosis in human glioma
Source: Discov Oncol. 2026 Jun 23;17:1169. doi: 10.1007/s12672-026-05494-z (PMC13469272; doi:10.1007/s12672-026-05494-z)
Supplement: Supplementary file 1 [file 12672_2026_5494_MOESM1_ESM.docx]

**Supplementary Figure 1. Calibration plots of the MRGS in TCGA and CGGA cohorts**

**(a)** and **(b)** Calibration plots at 3-year overall survival in the TCGA and CGGA cohorts, respectively, and **(c)** and **(d)** calibration plots at 5-year overall survival in the TCGA and CGGA cohorts, respectively. These plots illustrate the agreement between predicted and observed survival probabilities. Deviations from the ideal diagonal line were observed in both cohorts, indicating a tendency of the model to overestimate survival probabilities.
