# Supplementary material for: A novel mitochondrial-related gene signature for assessing the tumor immune microenvironment and predicting prognosis in human glioma
Source: Discov Oncol. 2026 Jun 23;17:1169. doi: 10.1007/s12672-026-05494-z (PMC13469272; doi:10.1007/s12672-026-05494-z)
Supplement: Supplementary file 2 [file 12672_2026_5494_MOESM2_ESM.docx]

**Supplementary Table S1.** R packages used in the study

| **Category** | **Package** | **Function in study** |
| --- | --- | --- |
| Data processing | Limma | Batch effect correction (removeBatchEffect) |
| Normalization | Limma | Data preprocessing / expression handling |
| Survival analysis | Surminer | Cox regression, Kaplan–Meier analysis |
| Feature selection | glmnet | LASSO Cox regression |
| Time-dependent ROC | timeROC | AUC calculation over time |
| Visualization | ggplot2 | Graphical representation |
| Heatmaps | Corrplot, HMISC | Heatmap generation |
| Immune analysis | Estimate | Stromal/immune score calculation |
| Immune deconvolution | CIBERSORT Web | Immune cell fraction estimation |
| Immune deconvolution | ImmuneCellAI R | T-cell subset estimation |
| Enrichment | GSEA 4.33 | Gene set enrichment analysis |
|  |  |  |

**Supplementary Table S1.** List of R packages used for data preprocessing, statistical analyses, immune deconvolution, and visualization in this study, provided to ensure transparency and reproducibility.
