# Supplementary material for: A novel mitochondrial-related gene signature for assessing the tumor immune microenvironment and predicting prognosis in human glioma
Source: Discov Oncol. 2026 Jun 23;17:1169. doi: 10.1007/s12672-026-05494-z (PMC13469272; doi:10.1007/s12672-026-05494-z)
Supplement: Supplementary file 3 [file 12672_2026_5494_MOESM3_ESM.docx]

**Supplementary Table S2**. List of 23 overlapping mitochondrial-related genes between TCGA and CGGA cohorts.

| Gene Symbol | *Full Name* |
| --- | --- |
| **B3GNT2** | Beta-1,3-N-Acetylglucosaminyltransferase 2 |
| **ALDH3A2** | Aldehyde Dehydrogenase 3 Family Member A2 |
| **ACSL1** | Acyl-CoA Synthetase Long Chain Family Member 1 |
| **FKBP10** | FK506 Binding Protein 10 |
| **LACTB** | Lactamase Beta |
| **STOML2** | Stomatin Like 2 |
| **PDK1** | Pyruvate Dehydrogenase Kinase 1 |
| **FDX1** | Ferredoxin 1 |
| **AREG** | Amphiregulin |
| **IMMT** | Inner Mitochondrial Membrane Protein |
| **MRPS5** | Mitochondrial Ribosomal Protein S5 |
| **FASTK** | Fas Activated Serine/Threonine Kinase |
| **P4HA1** | Prolyl 4-Hydroxylase Subunit Alpha 1 |
| **PGAM5** | Phosphoglycerate Mutase Family Member 5 |
| **BCL2A1** | BCL2 Related Protein A1 |
| **BAK1** | BCL2 Antagonist/Killer 1 |
| **ALKBH4** | AlkB Homolog 4, Fe(II) and 2-Oxoglutarate Dependent Dioxygenase |
| **NOL3** | Nucleolar Protein3 |
| **EFNB2** | Ephrin B2 |
| **NOX4** | NADPH Oxidase4 |
| **ACADVL** | Acyl-CoA Dehydrogenase Very Long Chain |
| **MRPL13** | Mitochondrial Ribosomal Protein L13 |
| **CDC25C** | Cell Division Cycle 25 |

**Supplementary Table S2**. Summary of the 23 mitochondrial-related genes identified as overlapping between the TCGA and CGGA glioma cohorts.
