# Supplementary material for: A novel mitochondrial-related gene signature for assessing the tumor immune microenvironment and predicting prognosis in human glioma
Source: Discov Oncol. 2026 Jun 23;17:1169. doi: 10.1007/s12672-026-05494-z (PMC13469272; doi:10.1007/s12672-026-05494-z)
Supplement: Supplementary file 4 [file 12672_2026_5494_MOESM4_ESM.docx]

**Supplementary Table S3.** Multivariate Cox proportional hazards analysis evaluating the independent prognostic value of the MTRS and clinical variables in TCGA cohort.

| Variables | Hazard ratios (HR) | 95% confidence intervals (CI) | p-values |
| --- | --- | --- | --- |
| Risk score | 2.17 | 1.44-3.3 | <0.01 |
| Age | 5.45 | 3.20-9.3 | <0.01 |
| Cancer type | 0.81 | 0.66-1.0 | 0.057 |
| Grade | 2.61 | 1.75-3.9 | <0.01 |
| Gender | 1.06 | 0.74-1.5 | 0.756 |

**Supplementary Table S3.** Hazard ratios (HR), 95% confidence intervals (CI), and corresponding p-values are reported for each variable included in the model. Clinical variables comprise age, gender, tumor grade, and cancer type (Histology). This analysis was conducted to assess whether the MRGS serves as an independent predictor of overall survival after adjustment for established prognostic factors.
