# Supplementary material for: A novel mitochondrial-related gene signature for assessing the tumor immune microenvironment and predicting prognosis in human glioma
Source: Discov Oncol. 2026 Jun 23;17:1169. doi: 10.1007/s12672-026-05494-z (PMC13469272; doi:10.1007/s12672-026-05494-z)
Supplement: Supplementary file 6 [file 12672_2026_5494_MOESM6_ESM.docx]

**Supplementary Table S5**. The information of the seven prognosis-related genes.

|  | Symbol | *Function* | *HR TCGA* | *P value TCGA* | *HR CGGA* | *P value CGGA* |
| --- | --- | --- | --- | --- | --- | --- |
| **ACADVL** | | ACADVL provides instructions for the production of an enzyme called very long-chain Acyl-CoA dehydrogenase (VLCAD). This enzyme functions mainly in mitochondria, and it’s crucial for fatty acid oxidation, a multi-step process that metabolizes fats and converts them into energy(1). | 2,153 | <0,0001 | 4,698 | <0,0001 |
|  | **FDX1** | A small iron-sulfur protein involved in the metabolism of various steroids hormones, cholesterol and bile acids, and transfers electrons from NADPH to mitochondrial cytochrome P450 via ferredoxin reductase (FDXR)(2). | 2,923 | <0,0001 | 0,6771 | <0,0001 |
|  | **ALDH3A2** | ALDH3A2 gene is a member of the aldehyde dehydrogenase (ALDH) gene family. It is responsible for the production of an enzyme called fatty aldehyde dehydrogenase (FALDH). This enzyme is implicated in the degradation of fats, in particular the breakdown of molecules called fatty aldehydes into fatty acids. This conversion is part fatty acid oxidation, during which the fats are broken down and converted into energy(3). | 1,458 | 0.0127 | 4,216 | <0,0001 |
|  | **ALKBH4** | -Dioxygenase engaged in the demethylation of monomethylated actin at 'Lys-84' (K84me1), serving as a regulator of actomyosin processes. Demethylation of actin K84me1 is required for the maintenance of actomyosin dynamics that allow normal cleavage furrow ingression during cytokinesis and cell migration(4) .  -Can be involved in transcription regulation (5). | 1,537 | 0,0018 | 2,933 | <0,0001 |
|  | **ACSL1** | Acsl1 is a key enzyme that drives fatty acids to the mitochondria for β-oxidation. The role of Acsl1 in energy metabolism has been unraveled in adipose tissue, heart and skeletal muscle (6). | 2,410 | <0,0001 | 2,771 | 0,0002 |
|  | **NOX4** | Nox is a distinct class of enzymes dedicated exclusively to the generation and regulation of oxidative stress in mitochondria*.* Nox-derived ROS are important in cellular physiology (7).  An increase in Nox expression and activation has been reported to enhance protein oxidation, resulting in impaired respiratory chain complex activity, altered mitochondrial function and bioenergetics, and enhanced oxidative stress (8). | 4,437 | <0,0001 | 6,646 | <0,0001 |
|  | **FASTK** | A mitochondria-specific isoform of FASTK is found in mitochondrial RNA granules and is involved in the biogenesis of ND6 mRNA, a subunit of the mitochondria-encoded NADH dehydrogenase complex (complex I) governing mitochondrial gene expression and function (9). | 2,149 | <0,0001 | 4,575 | <0,0001 |

**Supplementary Table S5.** Summary of the key characteristics of the seven prognosis-related genes identified as being significantly associated with glioma patient prognosis. For each gene, the hazard ratio (HR), and p-value in TCGA and CGGA are provided to show their individual prognostic significance.

**References**

1.	Tong MKH, Lam C-S, Mak TWL, Fu MYP, Ng S-H, Wanders RJA, Tang NLS. Very long-chain acyl-CoA dehydrogenase deficiency presenting as acute hypercapnic respiratory failure. *Eur Respir J* (2006) 28:447–450. doi: 10.1183/09031936.06.00139205

2.	Sun B, Ding P, Song Y, Zhou J, Chen X, Peng C, Liu S. FDX1 downregulation activates mitophagy and the PI3K/AKT signaling pathway to promote hepatocellular carcinoma progression by inducing ROS production. *Redox Biol* (2024) 75:103302. doi: 10.1016/j.redox.2024.103302

3.	Rizzo WB. Genetics and prospective therapeutic targets for Sjögren-Larsson Syndrome. *Expert Opin Orphan Drugs* (2016) 4:395–406. doi: 10.1517/21678707.2016.1154453

4.	Li M-M, Nilsen A, Shi Y, Fusser M, Ding Y-H, Fu Y, Liu B, Niu Y, Wu Y-S, Huang C-M, et al. ALKBH4-dependent demethylation of actin regulates actomyosin dynamics. *Nat Commun* (2013) 4:1832. doi: 10.1038/ncomms2863

5.	Kogaki T, Hase H, Tanimoto M, Tashiro A, Kitae K, Ueda Y, Jingushi K, Tsujikawa K. ALKBH4 is a novel enzyme that promotes translation through modified uridine regulation. *J Biol Chem* (2023) 299:105093. doi: 10.1016/j.jbc.2023.105093

6.	Kato A, Ito M, Sanaki T, Okuda T, Tsuchiya N, Yoshimoto R, Yukioka H. Acsl1 is essential for skin barrier function through the activation of linoleic acid and biosynthesis of ω-O-acylceramide in mice. *Biochimica et Biophysica Acta (BBA) - Molecular and Cell Biology of Lipids* (2022) 1867:159085. doi: 10.1016/j.bbalip.2021.159085

7.	Manea S-A, Constantin A, Manda G, Sasson S, Manea A. Regulation of Nox enzymes expression in vascular pathophysiology: Focusing on transcription factors and epigenetic mechanisms. *Redox Biol* (2015) 5:358–366. doi: 10.1016/j.redox.2015.06.012

8.	Vendrov AE, Vendrov KC, Smith A, Yuan J, Sumida A, Robidoux J, Runge MS, Madamanchi NR. NOX4 NADPH Oxidase-Dependent Mitochondrial Oxidative Stress in Aging-Associated Cardiovascular Disease. *Antioxid Redox Signal* (2015) 23:1389–1409. doi: 10.1089/ars.2014.6221

9.	Jourdain AA, Koppen M, Rodley CD, Maundrell K, Gueguen N, Reynier P, Guaras AM, Enriquez JA, Anderson P, Simarro M, et al. A mitochondria-specific isoform of FASTK is present in mitochondrial RNA granules and regulates gene expression and function. *Cell Rep* (2015) 10:1110–1121. doi: 10.1016/j.celrep.2015.01.063
